# Incidence and Risk Factors for Hypoglycemia During Fetal-to-Neonatal Transition in Premature Infants

**DOI:** 10.3389/fped.2020.00034

**Published:** 2020-02-11

**Authors:** Nikki A. Mitchell, Chelsey Grimbly, Elizabeth T. Rosolowsky, Megan O'Reilly, Maryna Yaskina, Po-Yin Cheung, Georg M. Schmölzer

**Affiliations:** ^1^Neonatal Research Unit, Centre for the Studies of Asphyxia and Resuscitation, Royal Alexandra Hospital, Alberta Health Services, Edmonton, AB, Canada; ^2^Medical School, University of Alberta, Edmonton, AB, Canada; ^3^Division of Pediatric Endocrinology, Department of Pediatrics, University of Alberta, Edmonton, AB, Canada; ^4^Women and Children's Health Research Institute, University of Alberta, Edmonton, AB, Canada; ^5^Division of Neonatology, Department of Pediatrics, University of Alberta, Edmonton, AB, Canada

**Keywords:** infant, newborn, hypoglycemia, glucose, diabetes, premature

## Abstract

**Objective:** To determine the incidence and risk factors associated with neonatal hypoglycemia in the premature population <33 weeks' gestation.

**Methods:** This was a secondary retrospective analysis from previous infants enrolled in randomized controlled trials. A total of 255 infants <33 weeks' gestation were born during the study period. Eight infants were excluded due to missing glucose or maternal data and 175 infants were analyzed.

**Main outcome measures:** Primary outcome was hypoglycemia (blood glucose <2.6mmol/L) determined via glucose oxidase method on arterial or venous blood gas. Birth weight subgroups: small for gestational age (SGA, birth weight <10%ile for gestational age) and large for gestational age (LGA, birth weight >90%ile for gestational age). Maternal hypertension was systolic blood pressure >140mmHg.

**Results:** 175 infants <33 weeks' gestational age (89 male, 84 female) were analyzed. Hypoglycemia occurred in 59 infants (33.7%). Maternal hypertension (OR 3.07, 95% CI 1.51–6.30, *p* = 0.002) was the sole risk factor for neonatal hypoglycemia. Protective factors for hypoglycemia included labor at time of delivery (OR 4.51, 95% CI 2.29–9.18, p <0.0001) and antenatal magnesium sulfate (OR 2.53, 95% CI 1.23–5.50, *p* = 0.01). There were no significant differences between hypoglycemic and euglycemic infants in sex, gestational age, LGA infants, antenatal steroids, vaginal birth, or maternal diabetes. SGA infants were excluded from analysis due to sample size.

**Conclusions:** Premature infants <33 weeks' gestation have increased risk of hypoglycemia. Maternal hypertension increases hypoglycemia risk. Antenatal magnesium sulfate administration or labor at time of delivery decrease hypoglycemia risk.

## Introduction

Neonatal hypoglycemia is a common occurrence during the first few days after birth as infants adjust to the extrauterine environment (1, 2). Hypoglycemia affects 5–10% of otherwise healthy infants, with increasing incidence reported in premature infants (1, 3). Defining neonatal hypoglycemia remains challenging, as infants can remain asymptomatic at even very low glucose concentrations or be symptomatic with even mild hypoglycemia. The Canadian Pediatric Society guidelines use the widely accepted definition of hypoglycemia as a blood glucose level of <2.6 mmol/L (1, 4–6).

The fetus does not accumulate glycogen until after 27 weeks' gestation, with a slow continual increase until after 36 weeks' gestation, and a rapid accumulation to reach 50 mg/g of tissue by term (7). After birth, the glucose concentration decreases to a nadir of 3–3.3 mmol/L in the first 1–2 h in term infants (2). Term infants use the stored glycogen for self-sufficient glucose homeostasis. In comparison, premature infants have lower glycogen stores and deplete them more quickly, putting them at higher risk for hypoglycemia after birth (7). They may also be exposed to more perinatal stress, leading to their premature delivery. There is a lack of data on the specific incidence and potential risk factors associated with hypoglycemia immediately after birth in premature infants. In late preterm or term infants, being born SGA or LGA, infants of diabetic mothers, perinatal stress including maternal hypertension are risk factors for neonatal hypoglycemia (4, 8, 9). The aim of the study was to determine incidence, risk factors and protective factors associated with neonatal hypoglycemia in premature infants <33 weeks' gestation.

## Methods

This was a secondary analysis from infants enrolled in previously published randomized controlled trials in premature infants (10, 11). The studies were carried out at The Royal Alexandra Hospital in Edmonton, Alberta, Canada, a tertiary perinatal center admitting more than 350 infants with a birth weight of <1,500 g annually. The Royal Alexandra Hospital Research Committee and Health Research Ethics Board, University of Alberta, approved the randomized controlled trials and subsequent collection of maternal data. Parental written consent was obtained to use the neonatal data. After our initial analysis, we observed that neonatal hypoglycemia was common in this study population, and we pursued analysis of the incidence and risk factors.

Inclusion criteria were neonates born <33 weeks' gestation between April 2013 and August 2014. Exclusion criteria included neonates with major congenital anomalies or conditions that might adversely affect breathing or ventilation (e.g., diaphragmatic hernia) as per previously published randomized controlled trials (10, 11). Deliveries were attended by the research team in addition to the Resuscitation-Stabilization-Triage team (RST-team) (neonatal nurse, respiratory therapist, nurse practitioner, and neonatal fellow). The research team was not involved in the clinical care of the infants. Delayed cord clamping was performed for 60 s if deemed appropriate by the obstetric team.

### Neonatal Demographics

Hypoglycemia was defined as blood glucose <2.6 mmol/L on venous or arterial blood gas sampling performed in the delivery room. Birthweight percentiles were calculated using the Fenton preterm growth chart (http://peditools.org/fenton2013/). SGA was defined as birth weight (BW) <10% for gestational age and LGA was defined as BW >90% for gestational age. Neonatal outcomes including necrotizing enterocolitis and intraventricular hemorrhage were recorded. All neonates were routinely fed based on local NICU protocols, and hypoglycemia was treated based on local NICU protocols.

### Maternal Demographics

Maternal Demographics included: (i) Maternal hypertension defined as a systolic blood pressure (SBP) >140 mmHg; (ii) Antenatal steroid administration divided into a) complete course of antenatal steroids (2 full doses of steroids before delivery of the infant), and b) receiving > one dose of steroids (defined as any antenatal steroids); iii) Antenatal magnesium sulfate (MgSO_4_) included all mothers who received MgSO_4_ prior to birth (administered as a 4 g loading dose, over 30 min, followed by a 1 g/h maintenance infusion until birth); (iv) Maternal diabetes was defined as diagnosis of (a) pre-gestational diabetes, or (b) gestational diabetes, defined as per the Diabetes Canada Clinical Practice Guidelines; (12) (v) labor (spontaneous or induced) at the time of delivery; and (vi) age at the time of delivery.

### Data Collection

The blood samples were collected from arterial or venous umbilical catheters into heparinized syringes and assayed immediately. Blood glucose concentrations were measured on blood gas samples collected within the first 90 min after delivery. The blood gas analyzer (ABL800 Flex; Radiometer Medical, Copenhagen, Denmark) used the glucose oxidase method (reading range 0.0–60 mmol/L, coefficient of variation 2.1%). Participant demographics and clinical information were collected along with maternal data including maternal age, medications, diabetes, and hypertension through review of hospital charts. Demographics of study infants were recorded in the database of the Center for the Studies of Asphyxia and Resuscitation.

### Statistical Analysis

The data are presented as mean with standard deviation (SD) for normally distributed continuous variables and median with interquartile range (IQR) when the distribution was skewed. Categorical data is presented by absolute and relative frequencies (n and %). Bivariate logistic regression models were performed to determine variables that significantly affect the outcome (infant hypoglycemia). Independent variables included sex, gestational age, birth weight, use of steroids, mode of delivery, labor at time of delivery, antenatal MgSO_4_, maternal SBP, and maternal diabetes. Variables with statistical significance of 0.15 and less and clinically significant variables (birth weight, use of steroids, labor at time of delivery, antenatal MgSO_4_, maternal SBP, and maternal diabetes) were included in the multivariable logistic regression model. Stepwise backward elimination was performed to obtain the final parsimonious model. Odds ratios are reported as a point estimate and 95% CI and *p* < 0.05 is considered as significant. All statistical analyses were performed by SAS 9.4 (SAS Institute Inc., Cary, NC).

## Results

A total of 255 infants <33 weeks' gestation born during the study period were enrolled; our analysis excluded 76 infants due to missing glucose measurements, and four additional infants were excluded due to missing maternal data. The demographics of the remaining 175 infants are presented in Table 1. The arterial or venous umbilical blood gas samples were analyzed at a median of 40 (IQR 30–55) minutes after birth, with no difference between hypoglycemic (median 47, IQR 31.5–58 min) or euglycemic infants (median 39, IQR 29–53 min). There was no sex (OR 0.92 (95%CI 0.49–1.73) *p* = 0.79) or gestational age differences (OR 1.09 (95% CI 0.96–1.24) *p* = 0.18) between hypoglycemic and euglycemic infants.

**Table 1:** Demographics of study infants.

	**Total**	**Hypoglycemic (*n* = 59)**	**Euglycemic (*n* = 116)**
**Infant**			
Male^*^	89^1^ (51.4%)	29 (49.2%)	60 (51.7%)
Gestational age (weeks)	28.2 (±2.5)	28.5 (±2.2)	28.0 (±2.7)
<28 weeks GA^*^	70 (40.0%)	23 (39.0%)	47 (40.5%)
28 ≤ 32 weeks GA^*^	88 (50.3%)	29 (49.2%)	59 (50.9%)
≥32 weeks GA^*^	17 (9.71%)	7 (11.9%)	10 (8.6%)
Singleton^*^	117 (67.6%)	42 (71.2%)	75 (64.7%)
**Birth weight**			
Birth weight (g)	1169 (±406)	1146 (±426)	1180 (±398)
BW percentile	56.20 (±27.63)	44.58 (±31.05)	61.65 (±24.14)
Birthweight Z score	0.23 (±1.03)	−0.05 (±1.41)	0.36 (±0.78)
SGA (BW <10%ile)^*^	9 (5.14%)	6 (10.2%)	3 (2.58%)
LGA (BW >90%ile)^*^	19 (10.9%)	6 (10.2%)	13 (11.2%)
**Apgar score**			
1 min^#^	5 (3-6)	5 (3-7)	4 (2-6)
5 min^#^	7 (6-8)	8 (6-9)	7 (6-8)
**Delivery**			
Vaginal delivery^*^	39 (23.4%)	8 (13.6%)	31 (26.7%)
Caesarian section^*^	128 (76.6%)	46 (78.0%)	82 (70.7%)
In labor^*^	97 (57.7%)	18 (30.5%)	79 (68.1%)
**Medications**			
Received antenatal ${\text{MgSO}}_{4}^{*}$	59 (35.5%)	12 (20.3%)	47 (40.5%)
Received antenatal steroids^*^	119 (72.1%)	41 (69.5%)	78 (67.2%)
**Blood gas**			
Time of ABG (age in minutes)	49.42 (±41.39) range 30–55	48.65 (±20.48) range 31.50–58	49.75 (±47.75) range 29–53
pH	7.19 (±0.13)	7.23 (±0.11)	7.18 (±0.13)
pCO_2_	60.99 (±19.11)	54.17 (±12.58)	64.14 (±20.78)
Lactate	3.76 (±2.86)	3.90 (±3.10)	3.69 (±2.75)
Base excess	−5.03 (±4.53)	−4.89 (±4.59)	−5.09 (±4.53)
**Maternal data**			
Age (years)	29.11 (±5.28)	29.64 (±5.23)	28.85 (±5.31)
Diabetes^*^	34 (21.3%)	14 (23.7%)	20 (17.2%)
Hypertension^*^	43 (24.6%)	23 (39.0%)	20 (17.2%)
Treated with ß-blockers	24 (55.8%)	11 (18.6%)	13 (11.2%)
Treated with other medications	14 (32.6%)	9 (15.3%)	5 (4.31%)
**Postnatal complications**			
Intraventricular hemorrhage^*^	49 (29.2%)	17 (28.8%)	32 (27.6%)
Necrotizing enterocolitis^*^	21 (12.9%)	6 (10.2%)	15 (12.9%)
Mortality^*^	17 (10.1%)	6 (10.2%)	11 (9.48%)

Data are presented as mean (SD) unless indicated

# median (IQR),]

* *n (%). GA = gestational age. SGA, small for gestational age. LGA, large for gestational age; BW, birth weight; MgSO_4_, magnesium sulfate; ABG, arterial blood gas*.

1 *Sex was undocumented in two infants*.

The overall incidence of hypoglycemia was 33.7% (*n* = 59). The risk factor associated with hypoglycemia was maternal hypertension (hypertension vs. normal: OR 3.07 (95%CI 1.51–6.30) *p* = 0.002), indicating a significantly greater proportion of hypoglycemia infants had hypertensive mothers. Protective factors for hypoglycemia, meaning a significantly greater proportion of infants were euglycemic than hypoglycemia, included being in labor at time of delivery (no vs. yes: OR 4.51 (95% CI 2.29–9.18) *p* < 0.0001), and administration of MgSO_4_ prior to delivery (no vs. yes: OR 2.53 (95%CI 1.23–5.50) *p* = 0.01). LGA (yes vs. no: OR 1.09 (95%CI 0.36–3.00) *p* = 0.87), vaginal birth (no vs. yes: OR 2.17 (95%CI 0.96–5.43) *p* = 0.08), antenatal steroids (no vs. yes: OR 0.67 (95%CI 0.31–1.41) *p* = 0.30), and maternal diabetes (no vs. yes: OR 0.66 (95%CI 0.31–1.47) *p* = 0.30) showed no significant difference between euglycemic and hypoglycemic infants. Significance of SGA infants was unable to be completed due to the small number of infants in this group. Results from both univariate and final models are presented in Table 2.

**Table 2:** Univariate and multivariate logistic regression.

	**Univariate analysis**			**Multivariate analysis**		
**Variable**	**Odds ratio**	**95% CI**	***p*-value**	**Odds ratio**	**95% CI**	***p*-value**
Sex (Male vs. Female)	0.92	0.49–1.73	0.79	Excluded		
Gestational age (in weeks)	1.09	0.96–1.24	0.18	Excluded		
SGA	Excluded—population too small			Excluded		
LGA (yes vs. no)	1.09	0.36–3.00	0.87	1.65	0.48–5.29	0.41
Antenatal Steroids (no vs. yes)	0.67	0.31–1.41	0.30	Excluded		
Vaginal delivery (no vs. yes)	2.17	0.96–5.43	0.08	Excluded		
Maternal diabetes (no vs. yes)	0.66	0.31–1.47	0.30	0.63	0.25–1.62	0.32
In labor at time of delivery (no vs. yes)	4.51	2.29–9.18	<0.0001	3.63	1.61–8.41	0.002
Antenatal MgSO_4_ (no vs. yes)	2.53	1.23–5.50	0.01	0.49	0.21–1.12	0.10
Maternal hypertension (HTN vs. normal)	3.07	1.51–6.30	0.002	2.40	1.003–5.71	0.047

*Data are presented as odds ratio for developing hypoglycemia with 95% confidence intervals. P-value <0.05 is considered significant. Variables with p < 0.11 and clinically significant variables were entered into multivariate logistical regression. Final model ROC, 0.75; SGA, small for gestational age; LGA, large for gestational age; MgSO_4_, magnesium sulfate; HTN, hypertension*.

## Discussion

To our knowledge, this is the first study to examine incidence and associated risk factors of hypoglycemia in premature infants <33 weeks' gestation within the first 90 min after birth. There is limited evidence regarding the specific incidence and risk factors associated with hypoglycemia in this population. Due to the design of this study, we have determined risk factors associated with neonatal hypoglycemia, but are not able to ascertain causality.

Our overall hypoglycemia incidence was ~34%, which is similar to previously reported data within the first day after birth in premature infants (13) and 1–48 h after birth in infants > 35 weeks. (1) James-Todd et al. reported an overall incidence of 41% in infants <32 weeks' gestation, however, they used point of care glucose measurements which have poor sensitivity in the hypoglycemia range and also excluded infants of diabetic mothers (13, 14). Harris et al. reported an incidence of 51% in infants ≥ 35 weeks' gestation within the first 48 h after birth (1). A recent study reported that the nadir of plasma glucose concentration in preterm infants and extremely preterm infants is after 70.5 and 60.9 min, respectively (15). Our glucose samples were taken at a mean postnatal age of 49.4 min, potentially missing the nadir of hypoglycemia and resulting in underestimation of our incidence.

Maternal hypertension was identified a risk factor for hypoglycemia, which increased the odds of hypoglycemia by 3.07 times. Maternal hypertension is a known risk factor for neonatal hypoglycemia and may reflect an environment of perinatal stress, placental insufficiency, risk factor for SGA, and risk factor for prematurity (9, 16, 17). In addition, maternal beta blocker exposure is a known risk factor for neonatal hypoglycemia (18). Beta blockers were the anti-hypertensive treatment used in over 55% of our population with maternal hypertension, which may have contributed to the increased risk of neonatal hypoglycemia.

MgSO_4_ administration was a protective factor for neonatal hypoglycemia in univariate analysis, but became non-significant in multivariate analysis. Antenatal MgSO_4_ reduces incidence of cerebral palsy and hemorrhage in infants born preterm, regardless of total dose (19–21). MgSO_4_ has been shown to decrease blood brain barrier permeability and increase plasma glucose concentration in a rat model of hypoglycemia (22). In addition, MgSO_4_ levels have been associated with lipoprotein metabolism and signal transduction in the insulin pathway (23–25). We hypothesized that MgSO_4_ decreased the utilization of glucose in the brain through a reduction in cerebral stress and blood brain barrier permeability. Combined with the impact of MgSO_4_ on appropriate insulin regulation and lipoprotein metabolism, we hypothesize that the protective mechanism of MgSO_4_ for maintenance of euglycemia is likely multifactorial.

Our results indicate that maternal labor may be protective against hypoglycemia in premature infants. Campbell et al. reported an increased release of endogenous steroids during labor in 80 women suggesting that these higher circulating endogenous steroids (i.e., cortisol) potentially initiate gluconeogenesis in infants during labor (26). This gluconeogenic environment of the fetus may be protective to neonates.

Antenatal steroid administration was neither a significant risk factor nor protective in our study population. Initial trials of steroid administration found no significant differences (27), and many studies do not include hypoglycemia data (28). However, studies report that antenatal steroid administration was associated with increased risk of neonatal hypoglycemia in late preterm infants born at 34–36 weeks of gestation (29). Administration of betamethasone for lung maturity in premature infants has been shown to suppress maternal adrenocorticotropic hormone and cortisol levels for up to 24 h, which could explain why antenatal steroid administration was not protective (30, 31).

LGA infants were not at increased risk for hypoglycemia in our population, and SGA infants were unable to be analyzed due to sample size; however, previously studies have demonstrated that these infants are at risk for hypoglycemia (8, 32–36). Infants born SGA have decreased glycogen and fat stores, inappropriate release of insulin, and impaired counter regulatory hormones, leading to increased risk of neonatal hypoglycemia (8, 34, 35). Infants born LGA have increased hyperinsulinism, leading to their excessive growth and inappropriate response to hypoglycemia antenatally (36, 37). Infants of diabetic mothers are more likely to be LGA and they have been previously reported to be associated with neonatal hypoglycemia, although this population was predominantly term infants (32, 33). Therefore, while LGA infants did not demonstrate significant increase in risk of hypoglycemia in our population, and significance of SGA infants was unable to be completed due to sample size. Further studies with a larger number of infants might yield different results.

Maternal diabetes was not a risk factor for hypoglycemia in our neonatal population. However, current guidelines recommend universal screening for gestational diabetes at 24–28 weeks' gestation, and ~53% of our infants were <28 weeks, when a large portion of the mothers might have had impaired glucose tolerance that was not yet diagnosed (33). Insulin requirements typically increase into the third trimester due to increasing hormonal levels leading to insulin resistance as well as pancreatic beta cell dysfunction (38, 39). Our population may not have yet been exposed to a high insulin and hormonally-induced insulin resistant environment, explaining why this was not a significant risk factor.

A limitation of our study was the retrospective analysis from prospectively collected data, therefore some important variables may have not been collected, including symptoms that occurred when infants were hypoglycemic. A second limitation was the smaller sample size which affected the statistical analysis of smaller populations and groups, most notably SGA infants. While two separate RCTs were combined for this study, they both occurred within an overlapping 16-month time period within the same hospital and with identical treatment protocols aside from the respiratory treatment intervention; therefore, no significant difference in treatment is likely to have affected hypoglycemia (10, 11). In addition, this study examined the transition phase immediately after birth. Our hospital policy dictates central venous access (i.e., umbilical venous access or peripheral venous access) as soon as possible after initial respiratory stabilization to provide continues dextrose infusion; therefore, delayed hypoglycemia, and persistent hypoglycemia were not addressed.

Future studies should examine blood glucose and insulin levels in mothers and insulin and ketone levels in infants during the fetal to neonatal period to potentially identify regulatory mechanisms, as well as long-term follow-up of the infants including neonatal complications and neurodevelopmental outcomes. In addition, investigations examining the mechanism of action between MgSO_4_ and neonatal hypoglycemia could provide insight into the development of preventative treatments.

## Conclusion

The incidence of hypoglycemia in premature infants <33 weeks' gestational age in our study was 33.7%. The risk factor associated with developing hypoglycemia was maternal hypertension, while antenatal administration of magnesium sulfate and being in labor at time of delivery were both protective factors. Due to the high incidence of hypoglycemia in the premature population, we recommend providing glucose infusion as soon as possible after birth, and continued screening and treatment as per local hospital guidelines.

## Data Availability Statement

All datasets generated for this study are included in the article/supplementary files.

## Ethics Statement

The Royal Alexandra Hospital Research Committee and Health Research Ethics Board, University of Alberta, approved the randomized controlled trials and subsequent collection of maternal data. Parental written consent was obtained to use the neonatal data. After our initial analysis, we observed that neonatal hypoglycemia was common in this study population, and we pursued analysis of the incidence and risk factors.

## Author Contributions

GS, MO, P-YC, CG, and MY contributed to conception and design. GS, MO, P-YC, CG, NM, and MY were involved in collection and assembly of data, analyzed and interpreted the data, and drafted the article. GS, MO, P-YC, CG, NM, MY, and ER contributed to critical revision of the article for important intellectual content and participated in final approval of the article.

### Conflict of Interest

The authors declare that the research was conducted in the absence of any commercial or financial relationships that could be construed as a potential conflict of interest.

## Abbreviations

BW: birth weight
GA: gestational age
IQR: interquartile range
LGA: large for gestational age
MgSO_4_: magnesium sulfate
RST: resuscitation-stabilization-triage team
SBP: systolic blood pressure
SD: Standard deviation
SGA: Small for gestational age.
